# Functional screening implicates miR-371-3p and peroxiredoxin 6 in reversible tolerance to cancer drugs

**DOI:** 10.1038/ncomms12351

**Published:** 2016-08-03

**Authors:** Nisebita Sahu, Jean-Philippe Stephan, Darlene Dela Cruz, Mark Merchant, Benjamin Haley, Richard Bourgon, Marie Classon, Jeff Settleman

**Affiliations:** 1 Department of Discovery Oncology, Genentech, 1 DNA Way, South San Francisco, California 94080, USA; 2 Department of Protein Chemistry, Genentech, 1 DNA Way, South San Francisco, California 94080, USA; 3 Department of Translational Oncology, Genentech, 1 DNA Way, South San Francisco, California 94080, USA; 4 Department of Molecular Biology, Genentech, 1 DNA Way, South San Francisco, California 94080, USA; 5 Department of Bioinformatics and Computational Biology, Genentech, 1 DNA Way, South San Francisco, California 94080, USA; 6 Department of Cancer Targets, Genentech, 1 DNA Way, South San Francisco, California 94080, USA

## Abstract

Acquired resistance to cancer drug therapies almost always occurs in advanced-stage patients even following a significant response to treatment. In addition to mutational mechanisms, various non-mutational resistance mechanisms have now been recognized. We previously described a chromatin-mediated subpopulation of reversibly drug-tolerant persisters that is dynamically maintained within a wide variety of tumour cell populations. Here we explore a potential role for microRNAs in such transient drug tolerance. Functional screening of 879 human microRNAs reveals miR-371-3p as a potent suppressor of drug tolerance. We identify *PRDX6* (peroxiredoxin 6) as a key target of miR-371-3p in establishing drug tolerance by regulating PLA2*/*PKCα activity and reactive oxygen species. *PRDX6* expression is associated with poor prognosis in cancers of multiple tissue origins. These findings implicate miR-371-3p as a suppressor of *PRDX6* and suggest that co-targeting of peroxiredoxin 6 or modulating miR-371-3p expression together with targeted cancer therapies may delay or prevent acquired drug resistance.

## Abstract (web summary)

Acquired resistance significantly limits the efficacy of cancer drug therapies. Here, the authors identify miR-371-3p as a suppressor of drug tolerance in cancer cell lines by its target gene PRDX6, which in turn regulates PLA2/PKCα signalling and ROS levels.

## Graphical abstract

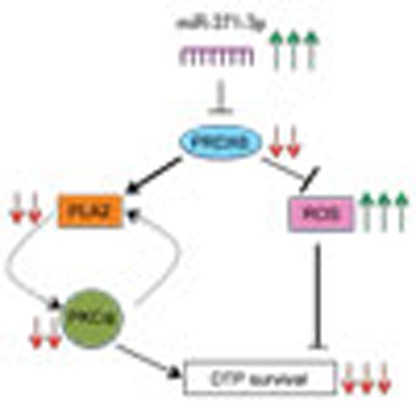

The relatively rapid emergence of resistance to cancer drug therapies is a major cause of treatment failure for patients^1^,^2^,^3^,^4^,^5^,^6^,^7^,^8^. Numerous mechanisms have now been identified that contribute to drug resistance, which include both mutational (genetic) and non-mutational (presumably epigenetic) mechanisms that ultimately render tumours unresponsive to a treatment that was initially effective^9^,^10^. Drug-tolerant persisters (DTPs) constitute a subpopulation of tumour cells that emerge at relatively high frequency upon treatment of largely drug-sensitive cancer cell populations with various anti-cancer agents^11^. DTPs are widely observed in various cancer contexts and exhibit a reversible ability to survive otherwise lethal drug exposures, implicating epigenetic regulation^1^,^2^,^9^,^10^.

Accumulating evidence suggests a role for microRNAs (miRNAs) in epigenetically regulating various phenotypic states in cancer cells^1^,^2^,^12^,^13^,^14^,^15^,^16^,^17^,^18^. miRNAs can impact genetic programs through post-transcriptional silencing of target genes either by promoting degradation of target messenger RNAs (mRNAs) or by inhibiting their translation^19^,^20^. miRNAs have been implicated in regulation of various aspects of cancer biology, including drug resistance, cancer cell stemness, epithelial-to-mesenchymal transition and metastasis^19^,^20^. We therefore hypothesize that miRNAs may also regulate genes required to engage the DTP state, and test this possibility by conducting a genome-wide functional screen of miRNAs to identify those with the ability to affect the establishment of the DTP state.

## Results

### Functional screening reveals miR-371-3p as a regulator of DTPs

**Figure 1:**
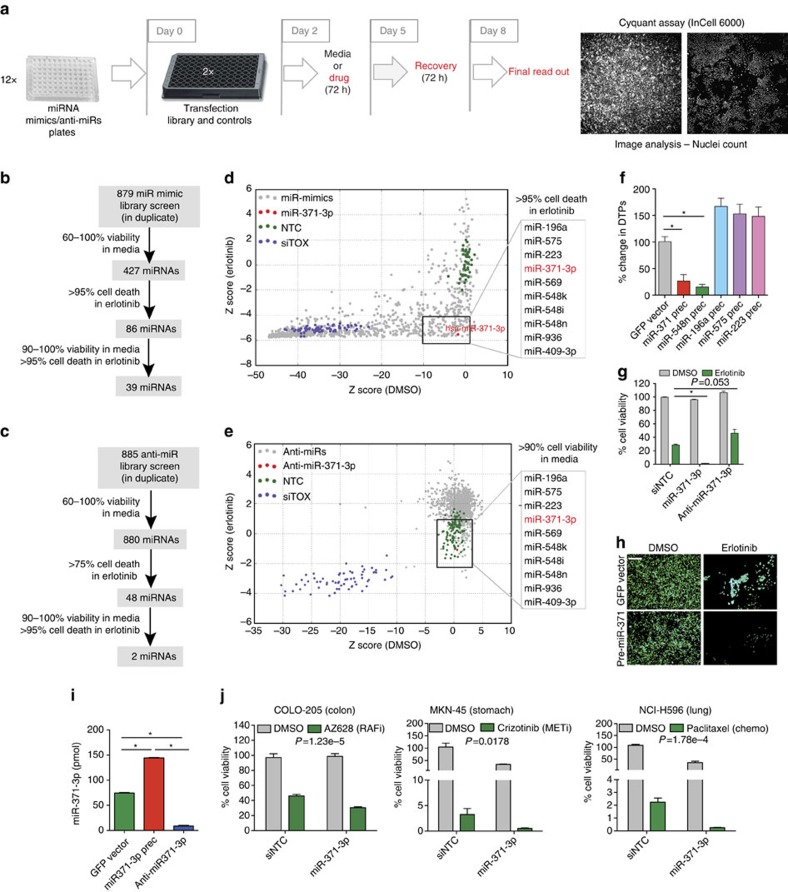
Functional screening identifies miR-371-3p as a regulator of DTPs. (**a**) miRNA mimic or inhibitor screening strategy. (**b**) miR-mimic library and (**c**) anti-miR library screen summary. (**d**) miR mimic screen data and (**e**) anti-miR screen data plotted as *z* scores of miRNAs in erlotinib (*y* axis) versus DMSO (*x* axis). (**f**) DTP count in miRNA mimic-expressing PC9 cells. (**g**) Effects of miR-371-3p, anti-miR371-3p and controls on DTPs. (**h**) Representative fluorescence images of GFP control or pre-miR-371-expressing parental cells and DTPs. Scale bar, 20 μm (**i**) Expression of miR-371-3p in a pre-miR-371-or anti-miR-371-expressing PC9 line validated by absolute quantitative PCR. (**j**) miR-371-3p overexpression reduces DTPs in COLO-205, MKN-45 and NCI-H596 cells upon drug treatment. All experiments were performed in triplicate and data are representative of at least two independent experiments. Data are represented as mean±s.e.m. For **f**, **g** and **i**, *P<0.05, Student's *t*-test. For **j**, *P* represents interaction *P* value.

To investigate whether miRNAs are required to engage or maintain the drug-tolerant state, we functionally screened 879 miRNA precursors and 885 miRNA inhibitors (anti-miRs) for cooperation with anti-cancer drugs to inhibit the emergence of DTPs. In a primary screen, *EGFR* mutant non-small cell lung cancer cells (PC9 cell line) were transfected with the miRNA libraries and cultured in the presence or absence of the EGFR kinase inhibitor erlotinib for 3 days, followed by a 3-day drug-free recovery phase to identify miRNAs that specifically affect DTPs (Fig. 1a). Sixty-seven per cent of miRNA precursors caused general cytotoxicity (<60% viability), even in the absence of drug, suggesting the requirement for a large number of miRNAs in critical biological control networks. Thirty-nine miRNAs (6%) significantly reduced the number of DTPs formed on erlotinib treatment without having any detectable effect on cell viability in the absence of the drug (Fig. 1b,d; Supplementary Fig. 1i; Supplementary Data 1). In contrast, 55% of the anti-miRs increased DTPs (Supplementary Fig. 1j). Only two of the anti-miRs significantly decreased DTPs (Fig. 1c,e; Supplementary Fig. 1j; Supplementary Data 2). However, overexpression of these miRNAs in the miR-mimic screen did not increase cell viability. Hence, further validation of five of the top hits from the miR mimic screen that caused >95% cell death upon overexpression and a 1.5–2-fold increase in cell viability upon inhibition was undertaken using stable PC9 cells overexpressing each miRNA precursor (pre-miRs).

Erlotinib-treated PC9 cells stably expressing pre-miRs revealed 2 miRNAs (pre-mir371 and pre-miR-548n) that substantially reduced DTPs without affecting the parental population (Fig. 1f; Supplementary Fig. 1a,c). The most robust effect was seen with pre-miR-371, which yields mature miR-371-3p and miR-371-5p isoforms (Fig. 1g–i; Supplementary Fig. 1b–d). Expression of miR-371-3p was decreased in DTPs in comparison with parental cells and in drug-tolerant expanded persisters (DTEPs), which eventually emerge following continuous exposure of cancer cells to appropriate drugs^11^, suggesting that miR-371-3p specifically plays an important role in the survival of DTPs (Supplementary Fig. 1e). Overexpressed pre-miR-371 caused similar effects in other cancer cell line models of different tissue origins with distinct drug sensitivities, implicating miR-371-3p in a widely used drug tolerance mechanism (Fig. 1j; Supplementary Data 3). Notably, some cell lines experienced cytotoxicity upon miR-371-3p overexpression in the absence of drug, possibly reflecting its importance in cell viability in the non-DTP fraction of some cancers.

### Identification of relevant miR-371-3p targets

**Figure 2:**
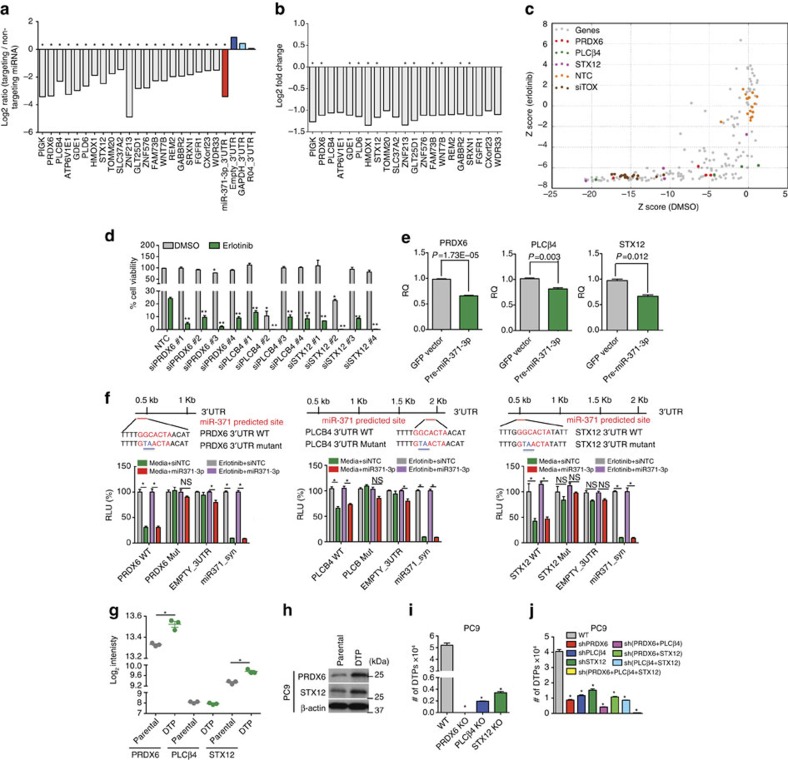
Identification of relevant miR-371-3p targets. (**a**) Downregulation of luciferase by miR-371-3p upon erlotinib treatment from 3′UTR reporters corresponding to candidate miR-371-3p target genes *P<0.05. (**b**) Microarray analysis of mRNA expression of miR-371-3p targets in pre-miR-371-expressing PC9 cells *P<0.05. (**c**) siRNA screen data corresponding to miR-371-3p targets plotted as *z* scores of siRNAs in erlotinib (*y* axis) versus DMSO (*x* axis)-treated cells. (**d**) Validation of siRNA knockdown effects of miR-371-3p targets, *PRDX6*, *PLCβ4* and *STX12* on DTPs from PC9 cells. * and ** indicate significant differences from DMSO- and erlotinib-treated NTC controls, respectively. (**e**) Quantitative PCR of gene expression of *PRDX6*, *PLCβ4* and *STX12* in pre-miR-371-expressing PC9 cells. (**f**) Mutations of seed sequences of the putative miR-371-3p recognition element in target 3′UTR's prevented inhibition by miR-371-3p. (**g**) *PRDX6*, *PLCβ4* and *STX12* mRNA, and (**h**) protein expression in PC9 parental cells and DTPs. (**i**) DTP count of PC9 cells with *PRDX6*, *PLCβ4* and *STX12* CRISPR/Cas9 knockouts, and (**j**) PC9 cells expressing sh*PRDX6*, sh*PLCβ4* and sh*STX12* single, double or triple knockdowns. * indicates significant differences from wild-type (WT) controls. All experiments were performed in triplicate and data are representative of at least two independent experiments. Data are represented as mean±s.e.m. * and **P<0.05, Student's *t*-test. NS, not significant.

Next, candidate gene targets of miR-371-3p were identified using the TargetScan prediction algorithm. The *KDM5A* and *IGF1R* genes, which encode proteins previously found to regulate DTPs, did not display any 3′-untranslated region (UTR) regions that could be potentially targeted by miR-371. Direct regulation by miR-371-3p of the top 70 predicted targets was assessed using a luciferase reporter assay following erlotinib treatment in the presence of miR-371-3p. In addition, *KDM-3B*, which displayed partial sequence homology in the 3′UTR with miR-371, was explored using the 3′UTR luciferase reporter assay. However, miR-371 was unable to target the 3′UTR of *KDM-3B*, suggesting that miR-371 does not regulate DTPs via *KDM-3B* (Supplementary Fig. 1h). Treatment with the histone deacetylase inhibitor trichostatin A also downregulated the expression of miR-371-3p, suggesting that miR-371-3p-mediated regulation of DTP formation is distinct from KDM5A/IGF-1R-mediated regulation (Supplementary Fig. 1c). Significantly, 21 genes displayed reduced luciferase activity upon erlotinib treatment, implicating these genes as bona fide targets of miR-371-3p (Fig. 2a). Microarray analysis of PC9 cells confirmed downregulation of only 12 of the predicted targets upon pre-miR-371 overexpression (Fig. 2b).

We then determined whether short interfering RNAs (siRNAs) directed against the 21 genes could mimic the effects of pre-miR-371 on DTPs (Fig. 2c; Supplementary Data 4). Transfection of si*PRDX6*, si*PLCβ4* or si*STX12* into PC9 cells decreased DTPs following erlotinib treatment without having an effect on cell viability in the absence of erlotinib treatment, reproducing the effects of pre-miR-371 (Fig. 2d; Supplementary Figs 2 and 3). These genes were also selectively downregulated by pre-miR-371 overexpression and upregulated by anti-miR-371 expression (Fig. 2e; Supplementary Fig. 1f). Mutation of the single putative miR-371-3p recognition site within *PRDX6*, *PLCβ4* and *STX12* 3′UTR sequences abolished the ability of miR-371-3p to inhibit luciferase reporter expression, confirming its direct regulation of these genes and suggesting that a single recognition element is sufficient for their regulation by miR-371-3p (Fig. 2f).

Gene expression analysis revealed increased expression of only *PRDX6* and *STX12* in DTPs (Fig. 2g,h; Supplementary Fig. 10); however, stable knockouts or knockdowns of *PRDX6*, *STX12* and *PLCβ4* genes in PC9 cells confirmed their requirement for DTP formation without affecting viability of the overall population in the absence of treatment (Fig. 2i,j; Supplementary Figs 4 and 5). Double knockdown of *PRDX6* and *PLCβ4*, and triple knockdown of *PRDX6*, *PLCβ4* and *STX12* produced the greatest effects on DTPs, implicating *PRDX6* and *PLCβ4* as the key mediators of miR-371-3p's function in drug tolerance (Fig. 2j; Supplementary Fig. 4). Indeed, knockdown of *PRDX6*, *PLCβ4* or *STX12* in five additional cancer cell lines similarly affected DTP formation (Supplementary Figs 2a and 3).

### Downregulation of PLA2/PKCα activity disrupts drug tolerance

**Figure 3:**
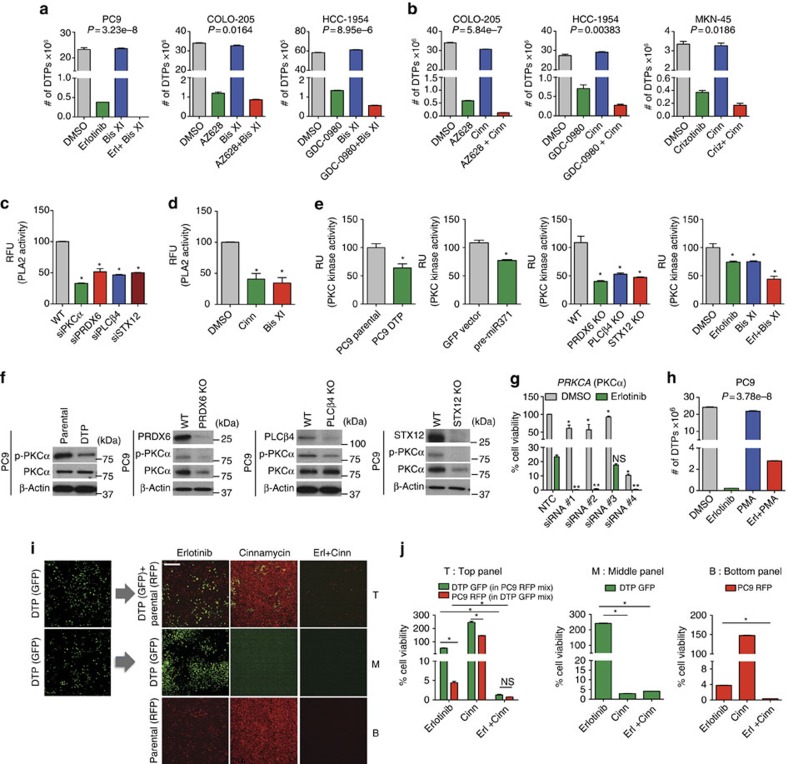
Downregulation of PRDX6/PLCβ4-associated PLA2/PKCα activity inhibits drug tolerance. (**a**) Co-treatment with bisindolylmaleimide XI reduces DTPs in PC9, COLO-205 and HCC-1954 cells. (**b**) Co-treatment with cinnamycin reduces DTPs in COLO-205, HCC-1954 and MKN-45 cells. Decreased PLA2 activity upon (**c**) siRNA knockdown of *PRDX6*, *PLCβ4* or *STX12* and (**d**) treatment with PLA2 inhibitors cinnamycin and the PKCα inhibitor bisindolylmaleimide XI. * indicates significant differences from wild-type (WT) or DMSO controls. (**e**) PKC activity in PC9 cells and DTPs, stable cell lines expressing pre-miR-371 or *PRDX6*, *PLCβ4* or *STX12* knockouts and upon treatment with bisindolylmaleimide XI±erlotinib. * indicates significant differences from DMSO or WT controls. (**f**) Immunoblot of PKCα activation in PC9 DTPs and PC9 cells with *PRDX6*, *PLCβ4* or *STX12* knockouts. (**g**) si*PRKCA* knockdown in PC9 cells reduces DTPs. * and ** indicate significant differences from DMSO- and erlotinib-treated NTC controls respectively. (**h**) Co-treatment with the PKC activator phorbol-12-myristate13-acetate (PMA) increases DTPs in PC9 cells **i**, Fluorescence microscopy images of surviving PC9 DTPs co-cultured with parental cells (T: top panel), PC9 DTPs (M: middle panel) or parental cells (B: bottom panel) after 6-day treatment with cinnamycin±erlotinib, Scale bar, 50 μm and (**j**) viable cell population counts of imaged cells in **f** on day 6 of treatment. All experiments were performed in triplicate and data are representative of at least two independent experiments. Data are represented as mean±s.e.m. For **c**–**e** and **g**, * or ** *P*<0.05, Student's *t*-test, NS, not significant. For **a**, **b** and **h**, *P* represents interaction *P* value.

Functional classification of the miR-371-3p targets revealed that four of the genes, including *PLCβ4* (phospholipase C, beta 4) and *PRDX6* (peroxiredoxin 6), encode proteins associated with phospholipase function^21^,^22^ (Supplementary Data 5). In contrast, *STX12* (syntaxin 12) is implicated in vesicle-mediated trafficking of EGFR and Src^23^. We therefore tested inhibitors of phospholipase activity as well as PKCα, a key component of phospholipase signalling. Phospholipase A2 (PLA2) inhibitors (cinnamycin and AACOCF3) and PKCα inhibitors (Bisindolylmaleimide I, IX, XI and PKC-20-28) reduced DTP formation (Fig. 3a,b; Supplementary Fig. 6a,b). PLA2 activity was also significantly reduced upon pre-miR-371 overexpression and upregulated by anti-miR-371 expression (Supplementary Fig. 1i). Knockdown of miR-371-3p targets, *PRDX6*, *PLCβ4* and *STX12*, as well as treatment with cinnamycin or bisindolylmaleimide XI, also decreased PLA2 activity (Fig. 3c,d). PKCα activity was reduced in the DTP subpopulation upon pre-miR-371 overexpression, depletion of *PRDX6*, *PLCβ4* and *STX12*, and following co-treatment with erlotinib and bisindolylmaleimide XI (Fig. 3e,f; Supplementary Fig. 10). Knockdown of *PRKCA* (PKCα) also decreased DTPs and downregulated PLA2 activity (Fig. 3a,g). Furthermore, DTP formation was increased upon treatment with the PKC agonist, phorbol 12-myristate 13-acetate, implicating PLA2/PKCα signalling as a key determinant of DTP formation (Fig. 3h). The decrease in PLA2 and PKCα activity on *STX12* knockdown was unexpected since its product, Syntaxin 12, has not been previously implicated in these pathways, and its specific function in this context remains unclear.

**Figure 4:**
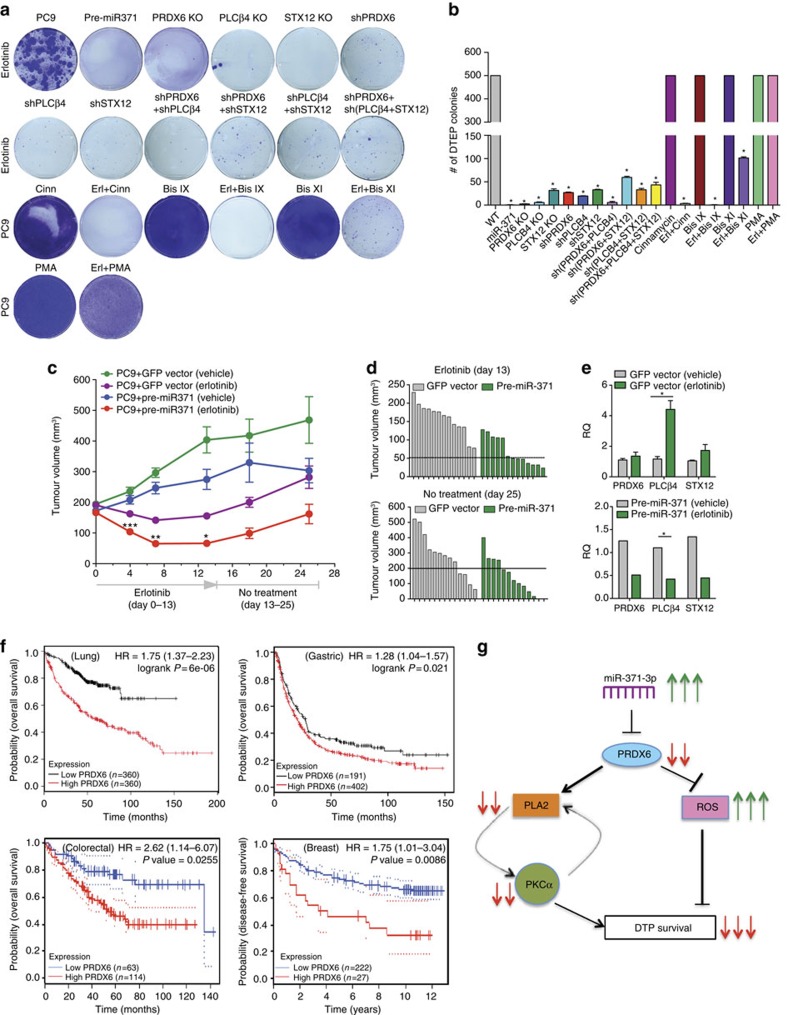
PRDX6-mediated PLA2/PKCα activity is required for drug tolerance. (**a**) DTEP formation after erlotinib treatment is reduced in PC9 cells on pre-miR-371 overexpression, single, double or triple knockdowns of *PRDX6*, *PLCβ4* or *STX12*, knockouts of *PRDX6*, *PLCβ4* or *STX12* and treatment with PLA2/PKCα inhibitors or phorbol-12-myristate13-acetate (PMA; *n*=3). (**b**) DTEP colony counts obtained in **a**. Data are representative of at least two independent experiments. Data are represented as mean±s.e.m. * (*P*<0.05, Student's *t*-test) indicate significant differences from wild-type (WT) controls. (**c**) Xenograft studies with PC9+GFP and PC9+pre-miR-371 cell lines using erlotinib (*n*=15). Data are represented as mean±s.e.m. Interaction *P* values *P=0.0288, **P=5.17e−05, ***P=0.00408. (**d**) Change in tumour volumes during drug treatment and after treatment cessation. (**e**) Quantitative PCR for expression of *PRDX6*, *PLCβ4* and *STX12* mRNAs in xenograft tumours (*n*=5). *P<0.05, Student's *t*-test. (**f**) Kaplan–Meier plots of overall survival of lung adenocarcinoma, colorectal cancer, gastric cancer and breast cancer patients; patient groups were separated based on *PRDX6* mRNA expression. (**g**) Model depicting proposed mechanism for regulation of DTP survival via miR-371-3p and *PRDX6*.

To determine whether PLA2 and PKCα inhibition targets the same DTP subpopulation within which expression of PRDX6 is upregulated, PC9-derived DTPs expressing GFP (DTP-GFP) were co-cultured with parental cells (PC9-RFP) in a 2.5:1 ratio and treated with cinnamycin or erlotinib and cinnamycin for 6 days. Cinnamycin reduced DTPs when cultured in the absence of parental cells, whereas viability of parental cells was unaffected (Fig. 3i,j). Co-treatment with erlotinib and cinnamycin reduced both populations under single as well as co-culture conditions. Sequential treatment with PLA2 or PKCα inhibitors (before or after erlotinib) demonstrated that co-treatment most effectively reduced DTPs, suggesting that dual inhibition of the EGFR and PLA2/PKCα signalling pathways is required for efficient targeting of both the parental and DTP population (Supplementary Fig. 6c,d). These inhibitors similarly affected DTPs in other tested cancer cell models (Fig. 3a,b). PLA2/PKCα inhibition also prevented the establishment of actively proliferating DTEPs (Fig. 4a,b). DTEP formation was also reduced upon stable miR-371 expression or following knockout or knockdown of *PRDX6*, *PLCβ4* and *STX12* (Fig. 4a,b).

In addition to its PLA2 activity, PRDX6 has a second function—regulation of oxidative stress via a peroxidase activity, and could potentially contribute to drug tolerance through this additional mechanism^21^,^24^,^25^,^26^. Notably, our previous studies demonstrated that increasing the levels of reactive oxygen species (ROS) selectively promotes cell death within the DTP subpopulation^27^. In PC9 cell lines expressing the three miR-371-3p target knockouts, as well as in DTP populations, ROS levels were decreased upon erlotinib treatment (Supplementary Fig. 7a,b). However, PC9 cells with stable knockout of *PRDX6* and *STX12* showed increased baseline ROS levels relative to parental and *PLCβ4* knockout cells, which were reduced in all of the lines upon erlotinib treatment (Supplementary Fig. 7b). Furthermore, low ROS levels were observed upon overexpression of miR-371-3p in the absence of drug treatment, but led to a significant increase in ROS levels on erlotinib treatment in comparison with the parental cell line (Supplementary Fig. 7c). These data further support our observations with knockout cell lines for miR-371-3p target genes, *PRDX6* and *STX12*, and suggest that miR-371-3p overexpression targets *PRDX6* and regulates ROS levels only upon cytotoxic drug treatment. Thus, increased baseline ROS levels due to the reduced PRDX6 and STX12 activity could contribute to increased cell death during drug treatment, further implicating ROS modulation in DTP survival.

### miR-371 overexpression enhances drug-induced tumour regression

We next assessed the role of pre-miR-371 overexpression in drug tolerance *in vivo* using PC9 and MKN-45 xenograft mouse models. Mice harbouring tumours derived from PC9+GFP vector and PC9+pre-miR-371 cell lines were treated with erlotinib. The growth rate of PC9 tumours was moderately reduced upon pre-miR-371 expression in the vehicle-treated group (Fig. 4c). Regression was observed in both PC9+GFP vector and PC9+pre-miR-371-derived tumours upon erlotinib treatment, but was significantly enhanced in the pre-miR-371-expressing tumours. After stopping erlotinib treatment for 12 days, tumour relapse was observed in 80% of mice harbouring PC9+GFP-derived tumours (Fig. 4c,d). In contrast, only 38% of mice with pre-miR-371-expressing tumours relapsed. Furthermore, expression of *PRDX6*, *PLCβ4* and *STX12* was upregulated in erlotinib-treated PC9+GFP tumours and was downregulated in erlotinib-treated PC9+pre-miR-371 tumours, suggesting that downregulation of these genes contributes to tumour regression and prevention of tumour regrowth during drug treatment (Fig. 4e). Similar effects were observed in mice harbouring tumours derived from MKN-45+pre-miR-371 cell lines, in contrast to MKN-45+GFP vector-bearing mice, upon treatment with the MET kinase inhibitor crizotinib and upon stoppage of treatment (Supplementary Fig. 2b,c). Notably, The Cancer Genome Atlas (TCGA) analysis of patient samples of several cancer types also revealed that relatively high expression of the miR-371-3p target *PRDX6*, but not *PLCβ4* or *STX12*, significantly correlated with poor prognosis in lung adenocarcinoma, colorectal, gastric and breast cancer patients (Fig. 4f; Supplementary Figs 8 and 9).

## Discussion

Together, these findings reveal the miR-371-3p target gene *PRDX6* as a key regulator of the reversible drug tolerance that frequently emerges within heterogeneous cancer cell populations. miR-371-3p's role in drug tolerance reflects its regulation of genes that encode components of both PLC/PKCα signalling and oxidative stress management. Recent studies have also implicated PKCα as the central regulatory node in breast cancer cells that are drug resistant and contribute to the emergence of more aggressive and treatment-refractory tumours^28^. The identification of PKCα as a regulator of drug tolerance implicates PKCα as a key driver of the emergence of drug-tolerant subpopulations of cancer cells. A PKC-dependent mechanism has also been recently reported to confer resistance to ALK inhibition in lung cancer^29^. In conclusion, our findings reveal a major regulatory role for miRNAs in the emergence of reversible drug tolerance, and suggest that combining current targeted therapies with inhibitors of PLA2/PKCα and ROS accumulation could provide a treatment strategy to delay some types of drug resistance in human cancer.

## Methods

### Cell lines and reagents

PC9 cells were from Kyushu University, Japan. EVSA-T and MKN-45 cells were from DSMZ. All other cell lines were from American Type Culture Collection. All cell lines were banked at the Genentech cell line core facility that routinely performs single nucleotide polymorphism (SNP) and short tandem repeats (STR) analysis to confirm cell line identity and detect possible mycoplasma contamination. PC9 cells were cultured in RPMI medium (Gibco) containing 4.5 g l^−1^ of D-glucose and supplemented with 10% fetal bovine serum, 2 mM L-glutamine, and 100 U ml^−1^ of penicillin and streptomycin. All other cell lines were maintained in RPMI medium (Gibco) supplemented with 10% fetal bovine serum, 2 mM L-glutamine, and 100 U ml^−1^ of penicillin and streptomycin. Lapatinib was from LC Laboratories, cinnamycin was from Sigma, AACOCF3 was from Santa Cruz Biotechnology, paclitaxel was from US Biologicals, carboplatin was from Selleckchem, AZ628 was from Tocris Bioscience, Bisindolylmaleimide I, XI and XI were from Cayman Chemicals, PKC-20-28 was from EMD Millipore, phorbol-12-myristate13-acetate was from VWR Scientific, and erlotinib and GDC-0980 were synthesized at Genentech.

### miRNA screen

PC9 cells were transfected with the Dharmacon miRIDIAN human miRNA mimic library (CS-001010; 879 miRNA mimics) or the Dharmacon miRIDIAN human miRNA inhibitor library (IH-001010; 885 anti-miRs) in 96-well plates. Reverse transfections were performed using Dharmafect 1 in duplicate with 12.5 nM of each miRNA or anti-miR. A total of 1,000 PC9 cells were plated per well. Forty-eight hours post transfection, media was replaced with media containing dimethylsulphoxide (DMSO) or 1 μM erlotinib and cells were incubated for 72 h. Drug-containing media was then removed and cells were cultured for another 72 h in drug-free medium, since DTPs were detectable in this short-term treatment regimen, which also enabled the robust quantification of viable drug-tolerant cells with good signal-to-noise ratio. Drug-tolerant cells were stained using the CyQUANT Direct Cell Proliferation Assay kit (Life Technologies) and cell counts were performed using an automated fluorescent microscope (In Cell Analyzer 6000; GE Healthcare). Cell viability for miR/anti-miRs-expressing cells was determined by calculating the ratio: (cell count in miR- or anti-miR-expressing cells)/(cell count in cells expressing non-targeting control (NTC) in DMSO). The *z* score (the number of s.d.'s from the mean) for each miRNA was calculated using Screensifter. Twenty-six of the top hits obtained from the screens were tested in eight additional cancer cell lines—COLO-205, MKN-45, NCI-H596, EVSA-T, M14, HCC-1954, NCI-H441 and SKBr3. COLO-205, EVSAT and HCC-1954 cells were transfected with RNAiMax, NCI-H596 and SKBr3 cells were transfected with Dharmafect 1, M14 cells were transfected with Dharmafect 3, and MKN-45 cells were transfected with Dharmafect 4. Drug treatments used for these cell lines were: COLO-205 (AZ628, 1 μM), MKN-45 (crizotinib, 1 μM), NCI-H596 (paclitaxel, 6 μM), EVSA-T (GDC-0980, 2.5 μM), M14 (AZ628, 1 μM), HCC-1954 (GDC-0980, 2.5 μM), NCI-H441 (carboplatin, 2.5 μM) and SKBr3 (lapatinib, 1 μM).

### 3′UTR luciferase reporter screen

Human 3′UTRs of potential targets for miR371-3p were selected from the LightSwitch genome-wide collection of 3′UTR luciferase reporters (Active Motif). A total of 89 3′UTR luciferase fusion reporters were selected from the genome-wide collection based on the presence of one or more predicted hsa-miR-371-3p target sites. An additional five reporters were used as negative controls, determined by a lack of predicted hsa-miR-371-3p target sites. The negative set included the empty vector, two 3′UTR constructs from housekeeping genes and two controls containing a random, non-genic, non-conserved sequence. A synthetic miRNA target sequence consisting of sequence repeats that are fully complementary to miR-371-3p and cloned downstream of the RenSP reporter gene in the pLightSwitch_3UTR vector was used as a positive control. Ninety-six-well plates were seeded with 15,000 PC9 cells 24 h before transfection to achieve 80% confluency at the time of transfection. Each transfection included 0.3 μl of DharmaFECT DUO transfection reagent (Dharmacon), 100 ng of 3′UTR reporter and mimic or non-targeting control miRNA (Dharmacon) to yield a final concentration of 50 nM in a total volume of 100 μl per well. Each construct was transfected in triplicate separately with either the miR-371-3p mimic or the non-targeting control. After 24 h, cells were treated with 1 μM erlotinib for an additional 24 h. A measure of 100 μl of LightSwitch Luciferase Assay Reagent (Active Motif) was then added to each well, plates were incubated at room temperature for 30 min and then read on a SpectraMax L luminometer (Molecular Devices). To identify genes that were significantly repressed, a *P* value (two-tail *t*-test) and log2-ratio was calculated for each reporter from the average luminescence values of the mimic and non-targeting control transfections.

### siRNA screen

A siRNA screen was performed following the same protocol as the miR screen using siRNAs corresponding to 21 hits short-listed from the 3′UTR luciferase reporter screen. Three or four single siRNAs per gene were obtained from Dharmacon for all the genes. Cell counts were performed after cells were stained with the CyQUANT kit using the In Cell Analyzer 6000. The *z* score (the number of standard deviations from the mean) for each siRNA was calculated using Screensifter. Cell viability was also measured at the end of the screen using the Cell Titer-Glo Luminescent Cell Viability Assay kit (Promega). Genes downregulated with two or more siRNAs and caused <40% cell death in the absence of drug treatment and >90% cell death on erlotinib treatment were considered for further analysis. The target sequences of si*PRKCA* (PKCα) used are as follows:

si*PRKCA* #1: GAAGGGTTCTCGTATGTCA

si*PRKCA* #2: GGACTGGGATCGAACAACA

si*PRKCA* #3: GGATTGTTCTTTCTTCATA

si*PRKCA* #4: TAAGGAACCACAAGCAGTA

### Site-directed mutagenesis

Mutations of seed sequences for miR-371-3p were generated using a modification of the QuikChange (Stratagene) protocol. Five nucleotides were mutated within a single target site in each 3′UTR reporter construct. After sequence confirmation, mutant reporters were tested along with wild-type controls in triplicate using the same protocol used for the 3′UTR luciferase reporter assay screen.

### Generation of DTPs and DTEPs

DTPs were generated by plating 10^6^ cells in 10 cm plates and after reaching 80% confluency, treating with 1 μM of relevant drugs for 9 days, replacing with fresh media containing drug every 72 h. The number of DTPs was determined using a Nexcelom Cellometer. DTEPs were generated by continuing the treatment regimen used for generating DTPs for 30 days. After 30 days, DTEP colonies were washed with PBS, and fixed and stained with 0.5% crystal violet containing 20% methanol. Individual DTEP colonies were counted and in cases where colonies were too numerous to count, they were given an arbitrary number of 500 colonies in the graphs plotted. All data represent mean±s.e.m. of three independent experiments.

### Immunofluorescence microscopy

PC9 cells stably expressing GFP were plated in six-well plates and treated with 1 μM erlotinib for 9 days. On day 9, the number of DTPs formed was counted by fluorescent microscopy using an In Cell Analyzer 6000. PC9 cells stably expressing RFP were then plated at a 2.5:1 ratio with DTPs. After 24 h, cells were treated with 1 μM erlotinib and/or cinnamycin for 6 days. After 6 days, surviving DTP and parental cells were counted by fluorescent microscopy using an In Cell Analyzer 6000.

### PKC activity assay

Cultured cells were treated for 24 h with relevant drugs and lysates were collected after 24 h. Cell lysates were prepared using kinase buffer (20–113, Millipore) containing protease inhibitor cocktail (ab65621, Abcam) and sonicated for 8 × 20 s intervals. Protein concentration was then quantified by BCA assay and 5 μg of protein lysates was used for each assay. The PKC activity assay was performed as per the manufacturer's instructions (ADI-EKS-420A, Enzo) and measured at an absorbance of 450 nm. Data were normalized to values from control, DMSO-treated cells.

### PLA2 activity assay

The PLA2 assay was conducted with cell lysates obtained using the same protocol described for the PKC activity assay. An amount of 5 μg of protein lysates was used and the assay was performed as per the manufacturer's instructions (E10217, Invitrogen). Fluorescence was measured at 450 nm using a SpectraMax M5 (Molecular Devices) and normalized to control cells.

### Cellular ROS detection assay

Cells were treated with DMSO or 1 μM erlotinib for 5 h and ROS levels were detected using the ROS detection agent, DCFDA, as per the manufacturer's instructions (ab113851, Abcam). Fluorescence was measured with excitation wavelength at 485 nm and emission wavelength at 535 nm using a SpectraMax M5 (Molecular Devices) and normalized to untreated PC9 parental cells. ROS levels in PC9 parental and DTP populations were measured by flow cytometry after performing the assay as per the manufacturer's instructions (C10491, Molecular Probes).

### Quantitative RT–PCR

For miRNA isolation, an RNA isolation kit (Exiqon) was used and complementary DNA was generated using a universal cDNA synthesis kit (Exiqon). PCR with reverse transcription (RT–PCR) was conducted using an ExiLENT SYBR Green Master Mix kit and LNA primer sets for miR-371-3p (cat #204299) as per the manufacturer's instructions. Absolute quantification of miR-371-3p was performed via standard curve method using synthetic oligonucleotides for miR-371-3p from Exiqon. RT–PCR was performed using an ABI Prism 7500 Real-Time PCR System as per the manufacturer's instructions.

For mRNA isolation, an RNAeasy kit (Qiagen) was used and RT–PCR was performed using Taqman RNA-to-C_T_ kit (Applied Biosystems) and Taqman probes from Applied Biosystems for *PRDX6* (Hs00705355_m1), *PLCβ4* (Hs00168656_m1), *STX12* (Hs00295291_m1) and *GAPDH* (Hs02758991_g1). RT–PCR was performed using an ABI Prism 7500 Real-Time PCR System. mRNA results were normalized to *GAPDH* and RQ was analysed using the ABI software.

### CRISPR/Cas9-mediated knockouts

Human codon optimized *S. pyogenes* Cas9 was cloned into a pRK vector and expressed via the human cytomegalovirus (CMV) immediate-early promoter. Individual sgRNAs were cloned downstream of and expressed from the human U6 promoter of the pLKO.5 vector (Sigma, product SHC-201). A total of 6 × 10^5^ cells were plated in six-well plates. After 24 h, cells were transfected with Cas9 and sgRNA constructs using Lipofectamine 2000. After 5 days of transfection, cells were selected with 2 μg ml^−1^ of puromycin for 24 h. Genomic DNA was isolated using a DNA Isolation kit (Qiagen) and deletion of the segment between each sgRNA pair was validated by performing PCR with locus-specific primers. Single-cell clones were obtained by sorting cells using a BD FACSAria Cell Sorter.

The following sgRNAs were used for generating knockouts in PC9 cells.


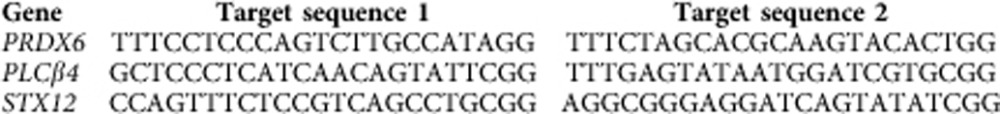


The following primers were used to validate CRIPSR/Cas9-mediated knockout in genomic DNA using PCR.


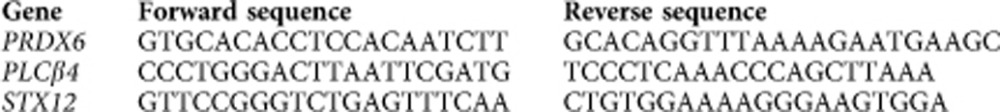


### Lentiviral infection

Lentiviral particles were generated as described previously^30^. For miRNA overexpression, human pre-miRNA Expression Constructs from System Biosciences that were used are: (Lenti-miR-371: PMIRH371PA-1), (Lenti-miR-196a: PMIRH196a1PA-1), (Lenti-miR-575: PMIRH575PA-1), (Lenti-miR-223: PMIRH223PA-1), (Lenti-miR-548n: PMIRH548nPA-1) and (Scramble control hairpin in pCDH-CMV-MCS-EF1-copGFP (CD511B-1): PMIRH000PA-1). For shRNA knockdowns, sh*PLCβ4* and sh*STX12* constructs in a lentiviral GFP vector were obtained from Origene, and sh*PRDX6* constructs were obtained from Genecopoeia.

### Protein expression analysis and antibodies

Cell lysates were prepared using RIPA lysis buffer (Thermo Scientific) containing a protease inhibitor cocktail (Thermo Scientific) and protein concentration was measured using the BCA assay (Thermo Scientific). An amount of 5 μg total protein was resolved by SDS–polyacrylamide gel electrophoresis and transferred to the nitrocellulose membrane (Bio-Rad). Immunoblotting was performed using 1:1,000 dilutions of the following antibodies, p-PKCα (sc-12356, Santa Cruz Biotechnology), PKCα (sc-208, Santa Cruz Biotechnology), PRDX6 (13585-1-AP, ProteinTech), PLCβ4 (sc-404, Santa Cruz Biotechnology), STX12 (Syntaxin 12) (sc-368438, Santa Cruz Biotechnology) and β-actin (3700S, Cell Signaling).

### Proliferation assays

Kinetic proliferation assays were performed by live-cell imaging using the IncuCYTE system (Essen Biosciences). Cells were plated in 96-well plates in triplicates and a proliferation time course was measured in the IncuCYTE for 72hrs. Data points were taken every 2 h.

### Bioinformatics

Predicted miR-371-3p target-binding sites were determined using TargetScan Human v.6.2. Gene functional classification for potential miR371-3p targets was performed using DAVID Bioinformatics Resources 6.7 (ref. 31; http://david.abcc.ncifcrf.gov/home.jsp).

### Statistical analysis

All data are represented as mean±s.e.m, unless otherwise specified. All replicates (*n*) were biological and represent two or three independent experiments. Interaction testing was performed for assessing interactions between miRNA and drug (Fig. 1i and fixed time points in Fig. 4c) or between two drugs (Fig. 3h–j). Standard linear models to regress log-scale response were used against the two main effects plus their interaction. The Wald statistic associated with the interaction term was used for an interaction *P* value. For all other figures, Student's *t*-test (two-tailed) was used to compare two groups and to calculate *P* values. *P*<0.05 was considered significant. All data were normally distributed and the variances were similar between the groups being statistically compared. The sample size for *in vitro* studies was based on the previous experience with experimental variability and no statistical method was used to predetermine the sample size. For *in vivo* xenograft studies, 10–15 tumour-bearing mice per experimental or control cohort were used based on power analyses that called for an *n* of 7 or greater to achieve a confidence level of 90% as per the variation in tumour volume between different groups and s.d. of tumour volumes within each group. No samples were excluded from the analysis. The investigators were not blinded to the group allocation during the experiment or for outcome assessment.

### Xenograft studies

All studies involving animals complied with protocols approved by the Institutional Animal Care and Use Committee at Genentech and all studies were carried out in an Association for the Assessment and Accreditation of Laboratory Animal Care-accredited facility. For PC9+GFP vector and PC9+pre-miR371 xenografts, 5 × 10^6^ cells were implanted subcutaneously into the right flanks of 6–8-week-old female Harlan Athymic Nude mice. For MKN-45+GFP vector and MKN-45+pre-miR371 xenografts, 10 × 10^6^ cells were implanted subcutaneously into the right flanks of 6–8-week-old female NCR Athymic Nude mice. Mice were randomized into two groups after tumours reached ∼200 mm^3^ and treated for 13 days with vehicle (MCT) or erlotinib (50 mg kg^−1^) for PC9 xenografts, and with vehicle (30% PEG400/0.5% Tween 80/5% propylene glycol) or crizotinib (50 mg kg^−1^) for MKN-45 xenografts. Drug treatment was then stopped for the next 12 days (days 13–25). Tumour sizes were measured throughout the study and graphed in the figures without any exclusion of samples. The investigators were not blinded to the group allocation during the experiment or for outcome assessment. At the end of the study, RNA was extracted from tumours using the RNeasy Plus kit from Qiagen as per the manufacturer's instructions.

### Kaplan–Meier survival curves

Kaplan–Meier survival analysis of patient samples was generated either using Kaplan–Meier plotter tool^32^ (http://kmplot.com) or PrognoScan^33^. The patients were divided into high versus low expressors based on the median value for each gene.

### Microarray analysis

For gene expression microarray analysis, PC9+GFP vector and PC9+pre-miR371-expressing stable cell lines were treated with DMSO or 2 μM erlotinib for 24 h and RNA was extracted using the RNeasy kit (Qiagen). For microarray analysis of DTPs, RNA was extracted from PC9+GFP vector treated with DMSO or 2 μM erlotinib for 9 days using the RNA isolation kit (Exiqon). Gene expression profiling was performed using the Affymetrix Hgu133plus2 platform. Gene expression data were normalized by quantile normalization^34^ and differentially expressed genes were identified using the LIMMA package, with false discovery rate and *P* value limited to <0.05 and fold changes >1.5.

### Data availability

Data generated during this study are available within the article, its Supplementary Information files. All microarray data generated in this study have been deposited in GEO with accession no. GSE83122.

## Additional information

**Accession codes:** Gene Expression Omnibus, GSE83122.

**How to cite this article**: Sahu, N. *et al*. Functional screening implicates miR-371-3p and peroxiredoxin 6 in reversible tolerance to cancer drugs. *Nat. Commun.* 7:12351 doi: 10.1038/ncomms12351 (2016).

## Supplementary Material

Supplementary Figures Supplementary Figures 1-10

Supplementary Data 1 miR mimic screen hits in PC9 cells

Supplementary Data 2 Anti-miR screen hits in PC9 cells

Supplementary Data 3 miR mimic screen validation in 8 cancer cell lines

Supplementary Data 4 siRNA screen of miR-371 targets

Supplementary Data 5 Gene functional classification of miR-371-3p target genes

## References

[b1] Holohan C., Van Schaeybroeck S., Longley D. B. & Johnston P. G. Cancer drug resistance: an evolving paradigm. Nat. Rev. Cancer 13, 714–726 (2013). 24060863 10.1038/nrc3599

[b2] Engelman J. A. & Settleman J. Acquired resistance to tyrosine kinase inhibitors during cancer therapy. Curr. Opin. Genet. Dev. 18, 73–79 (2008). 18325754 10.1016/j.gde.2008.01.004

[b3] Cara S. & Tannock I. F. Retreatment of patients with the same chemotherapy: implications for clinical mechanisms of drug resistance. Ann. Oncol. 12, 23–27 (2001). 11249045 10.1023/a:1008389706725

[b4] Kurata T. . Effect of re-treatment with gefitinib ('Iressa', ZD1839) after acquisition of resistance. Ann. Oncol. 15, 173–174 (2004). 14679138 10.1093/annonc/mdh006

[b5] Yano S. . Retreatment of lung adenocarcinoma patients with gefitinib who had experienced favorable results from their initial treatment with this selective epidermal growth factor receptor inhibitor: a report of three cases. Oncol. Res. 15, 107–111 (2005). 16119008

[b6] Lee H. J. . Drug resistance via feedback activation of Stat3 in oncogene-addicted cancer cells. Cancer Cell 26, 207–221 (2014). 25065853 10.1016/j.ccr.2014.05.019

[b7] Diaz L. A. Jr. . The molecular evolution of acquired resistance to targeted EGFR blockade in colorectal cancers. Nature 486, 537–540 (2012). 22722843 10.1038/nature11219 PMC3436069

[b8] Wheeler D. L., Dunn E. F. & Harari P. M. Understanding resistance to EGFR inhibitors-impact on future treatment strategies. Nat. Rev. Clin. Oncol. 7, 493–507 (2010). 20551942 10.1038/nrclinonc.2010.97 PMC2929287

[b9] Glasspool R. M., Teodoridis J. M. & Brown R. Epigenetics as a mechanism driving polygenic clinical drug resistance. Br. J. Cancer 94, 1087–1092 (2006). 16495912 10.1038/sj.bjc.6603024 PMC2361257

[b10] Trumpp A. & Wiestler O. D. Mechanisms of Disease: cancer stem cells--targeting the evil twin. Nat. Clin. Pract. Oncol. 5, 337–347 (2008). 18431377 10.1038/ncponc1110

[b11] Sharma S. V. . A chromatin-mediated reversible drug-tolerant state in cancer cell subpopulations. Cell 141, 69–80 (2010). 20371346 10.1016/j.cell.2010.02.027 PMC2851638

[b12] Lujambio A. & Lowe S. W. The microcosmos of cancer. Nature 482, 347–355 (2012). 22337054 10.1038/nature10888 PMC3509753

[b13] Migliore C. & Giordano S. Resistance to targeted therapies: a role for microRNAs? Trends Mol. Med. 19, 633–642 (2013). 24012193 10.1016/j.molmed.2013.08.002

[b14] Song S. J. . MicroRNA-antagonism regulates breast cancer stemness and metastasis via TET-family-dependent chromatin remodeling. Cell 154, 311–324 (2013). 23830207 10.1016/j.cell.2013.06.026 PMC3767157

[b15] Ma L., Teruya-Feldstein J. & Weinberg R. A. Tumour invasion and metastasis initiated by microRNA-10b in breast cancer. Nature 449, 682–688 (2007). 17898713 10.1038/nature06174

[b16] Calin G. A. . Frequent deletions and down-regulation of micro- RNA genes miR15 and miR16 at 13q14 in chronic lymphocytic leukemia. Proc. Natl Acad. Sci. USA 99, 15524–15529 (2002). 12434020 10.1073/pnas.242606799 PMC137750

[b17] Lu J. . MicroRNA expression profiles classify human cancers. Nature 435, 834–838 (2005). 15944708 10.1038/nature03702

[b18] Kasinski A. L. & Slack F. J. Epigenetics and genetics. MicroRNAs en route to the clinic: progress in validating and targeting microRNAs for cancer therapy. Nat. Rev. Cancer 11, 849–864 (2011). 22113163 10.1038/nrc3166 PMC4314215

[b19] Bartel D. P. MicroRNAs: genomics, biogenesis, mechanism, and function. Cell 116, 281–297 (2004). 14744438 10.1016/s0092-8674(04)00045-5

[b20] Bartel D. P. MicroRNAs: target recognition and regulatory functions. Cell 136, 215–233 (2009). 19167326 10.1016/j.cell.2009.01.002 PMC3794896

[b21] Rolfs F. . Dual role of the antioxidant enzyme peroxiredoxin 6 in skin carcinogenesis. Cancer Res. 73, 3460–3469 (2013). 23576553 10.1158/0008-5472.CAN-12-4369

[b22] Lyon A. M. & Tesmer J. J. Structural insights into phospholipase C-beta function. Mol. Pharmacol. 84, 488–500 (2013). 23880553 10.1124/mol.113.087403 PMC3781385

[b23] Williams K. C. & Coppolino M. G. SNARE-dependent interaction of Src, EGFR and beta1 integrin regulates invadopodia formation and tumor cell invasion. J. Cell. Sci. 127, 1712–1725 (2014). 24496451 10.1242/jcs.134734

[b24] Wang Y., Feinstein S. I. & Fisher A. B. Peroxiredoxin 6 as an antioxidant enzyme: protection of lung alveolar epithelial type II cells from H2O2-induced oxidative stress. J. Cell. Biochem. 104, 1274–1285 (2008). 18260127 10.1002/jcb.21703 PMC4922305

[b25] Sundar I. K. . Peroxiredoxin 6 differentially regulates acute and chronic cigarette smoke-mediated lung inflammatory response and injury. Exp. Lung. Res. 36, 451–462 (2010). 20939758 10.3109/01902141003754128 PMC3062974

[b26] Zhang S. . Protein and miRNA profiling of radiation-induced skin injury in rats: the protective role of peroxiredoxin-6 against ionizing radiation. Free Radic. Biol. Med. 69, 96–107 (2014). 24447893 10.1016/j.freeradbiomed.2014.01.019

[b27] Raha D. . The cancer stem cell marker aldehyde dehydrogenase is required to maintain a drug-tolerant tumor cell subpopulation. Cancer Res. 74, 3579–3590 (2014). 24812274 10.1158/0008-5472.CAN-13-3456

[b28] Tam W. L. . Protein kinase C alpha is a central signaling node and therapeutic target for breast cancer stem cells. Cancer Cell 24, 347–364 (2013). 24029232 10.1016/j.ccr.2013.08.005 PMC4001722

[b29] Wilson F. H. . A functional landscape of resistance to ALK inhibition in lung cancer. Cancer Cell 27, 397–408 (2015). 25759024 10.1016/j.ccell.2015.02.005 PMC4398996

[b30] Moffat J. . A lentiviral RNAi library for human and mouse genes applied to an arrayed viral high-content screen. Cell 124, 1283–1298 (2006). 16564017 10.1016/j.cell.2006.01.040

[b31] Huang, da W., Sherman B. T. & Lempicki R. A. Systematic and integrative analysis of large gene lists using DAVID bioinformatics resources. Nat. Protoc. 4, 44–57 (2009). 19131956 10.1038/nprot.2008.211

[b32] Gyorffy B. . An online survival analysis tool to rapidly assess the effect of 22,277 genes on breast cancer prognosis using microarray data of 1,809 patients. Breast Cancer Res. Treat. 123, 725–731 (2010). 20020197 10.1007/s10549-009-0674-9

[b33] Mizuno H., Kitada K., Nakai K. & Sarai A. PrognoScan: a new database for meta-analysis of the prognostic value of genes. BMC Med. Genomics 2, 18 (2009). 19393097 10.1186/1755-8794-2-18 PMC2689870

[b34] Smyth G. K. Linear models and empirical bayes methods for assessing differential expression in microarray experiments. Stat. Appl. Genet. Mol. Biol. 3, Article3 (2004). 16646809 10.2202/1544-6115.1027

