# Supplementary material for: Functional screening implicates miR-371-3p and peroxiredoxin 6 in reversible tolerance to cancer drugs
Source: Nat Commun. 2016 Aug 3;7:12351. doi: 10.1038/ncomms12351 (PMC4976141; doi:10.1038/ncomms12351)
Supplement: Supplementary Figures — 1-10 [file ncomms12351-s1.pdf]

Supplementary Information

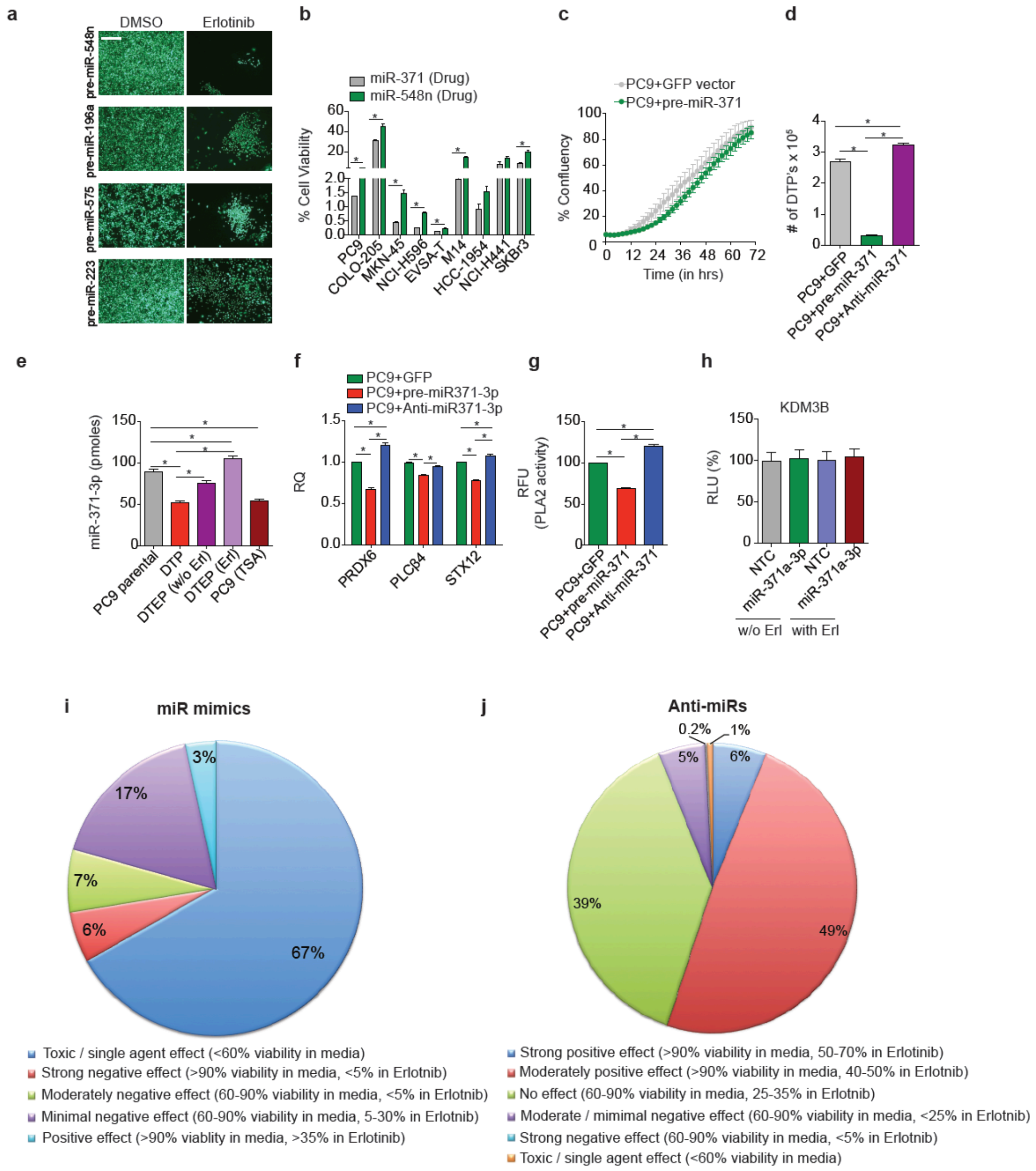

Supplementary Figure 1. Effect of miR mimics and anti-miRs on DTPs **a,** Representative fluorescence microscopy images of GFP vector control or miR mimic-expressing parental and DTP cells. Scale bar: 20μm **b,** Comparison of miR-371-3p and miR-548n on DTPs. **c,** Effect of pre-miR-371 overexpression on proliferation rate of PC9 stable lines analyzed by Incucyte. **d,** DTP count of PC9 cells expressing miR-371-3p, anti-

miR371-3p or controls. **e,** Expression of miR-371-3p in PC9 parental, DTP and DTEP populations validated by absolute qPCR. **f,** QPCR of gene expression of *PRDX6*, *PLCβ4* and *STX12* in pre-miR-371 and Anti-miR-371-expressing PC9 cells. **g,** PLA2 activity in pre-miR-371 and Anti-miR-371-expressing PC9 cells. **h,** regulation of luciferase by miR-371-3p upon erlotinib treatment from a 3′ UTR reporter of the *KDM3B* gene. **i,** The percent distribution of miR mimics corresponding to the full library screen based on their effect on viability of PC9 cells and DTP formation. **j,** The percent distribution of anti-miRs corresponding to the full library screen based on their effect on viability of PC9 cells and DTP formation. All experiments were performed in triplicate and data are representative of at least two independent experiments. Data are represented as mean ± S.E.M. *$P < 0.05$, Student's t-test.

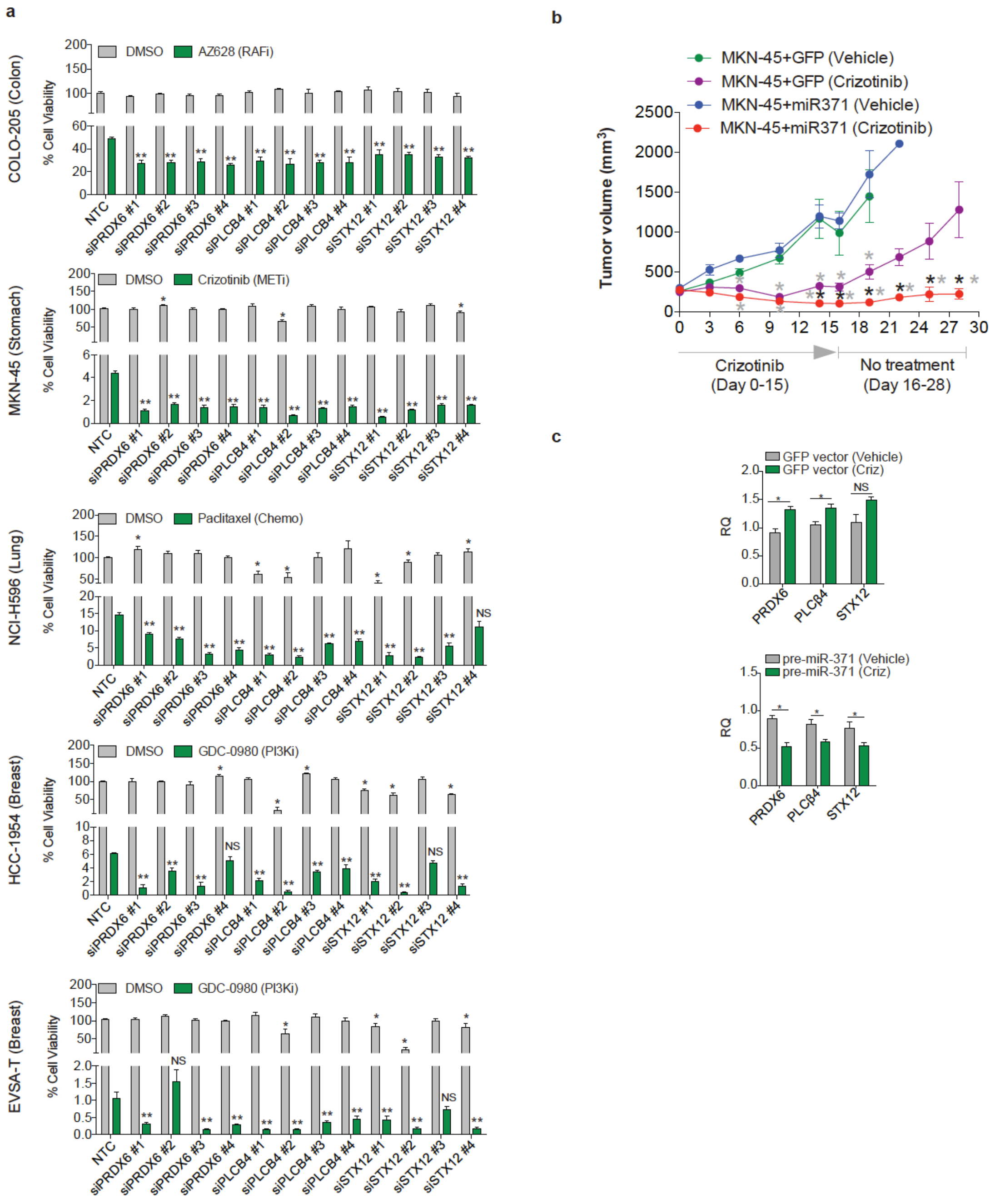

Supplementary Figure 2. Effect of *PRDX6*, *PLCβ4* and *STX12* knockdowns on DTP formation from cancer cells of different tissue origins a, DTP formation of COLO-205 (colon), MKN-45 (stomach), NCI-H596 (lung) , HCC-1954 (breast) and EVSA-T (breast)

cells transfected with siRNAs targeting *PRDX6*, *PLCβ4* or *STX12* was measured by Cell Titer-Glo after treatment with AZ628 (RAF kinase inhibitor), crizotinib (MET kinase inhibitor), paclitaxel (anti-mitotic chemotherapy), GDC-0980 (dual MTOR, PI-3 kinase inhibitor) treatments, respectively, for 72hrs, followed by a recovery phase of 72hrs. * and ** indicate significant differences from DMSO and drug-treated NTC controls, respectively. All experiments were performed in triplicate and data are representative of at least two independent experiments. **b,** Xenograft studies with MKN-45+GFP and MKN-45+pre-miR-371 lines using crizotinib (n=10). * and * indicate significant differences from vehicle and drug treated MKN-45+GFP tumor controls respectively. **c,** QPCR analysis of *PRDX6*, *PLCβ4* and *STX12* mRNA expression in MKN-45 xenograft tumors (n=5). Data are represented as mean ± S.E.M. Data are represented as mean ± S.E.M. * or ** *P*< 0.05, Student's t-test. NS, not significant.

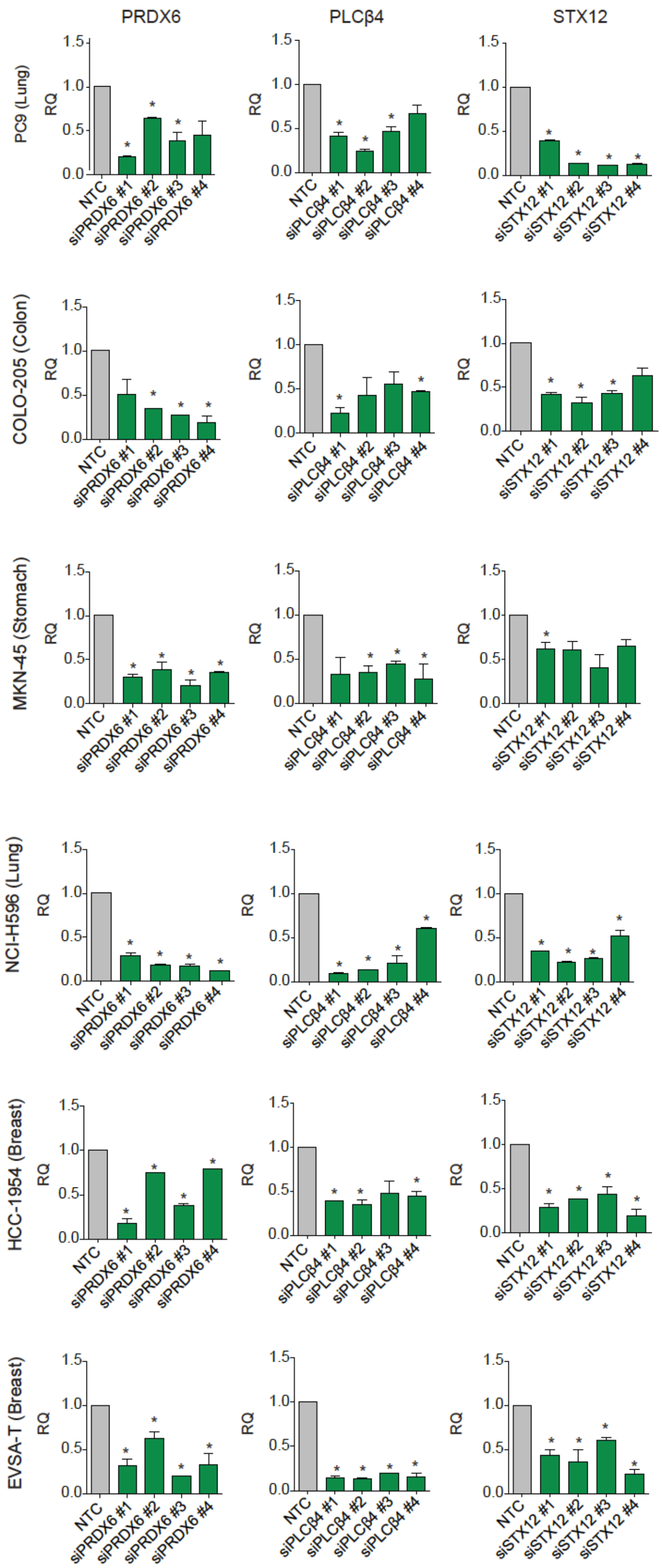
PRDX6
PLCβ4
STX12
PC9 (Lung)
COLO-205 (Colon)
MKN-45 (Stomach)
NCI-H596 (Lung)
HCC-1954 (Breast)
EVSA-T (Breast)
RQ
0.0
0.5
1.0
1.5
NTC
siPRDX6 #1
siPRDX6 #2
siPRDX6 #3
siPRDX6 #4
siPLCβ4 #1
siPLCβ4 #2
siPLCβ4 #3
siPLCβ4 #4
siSTX12 #1
siSTX12 #2
siSTX12 #3
siSTX12 #4
*

Supplementary Figure 3. Validation of siRNA knockdown by RT-PCR Quantitative PCR validation of siRNA knockdown efficiency of *PRDX6*, *PLCβ4* or *STX12* after 72hrs of transfection. All experiments were performed in triplicate and data are representative of at least two independent experiments. Data are represented as mean ± S.E.M. * ($P < 0.05$, Student's t-test) indicates significant differences from NTC controls.

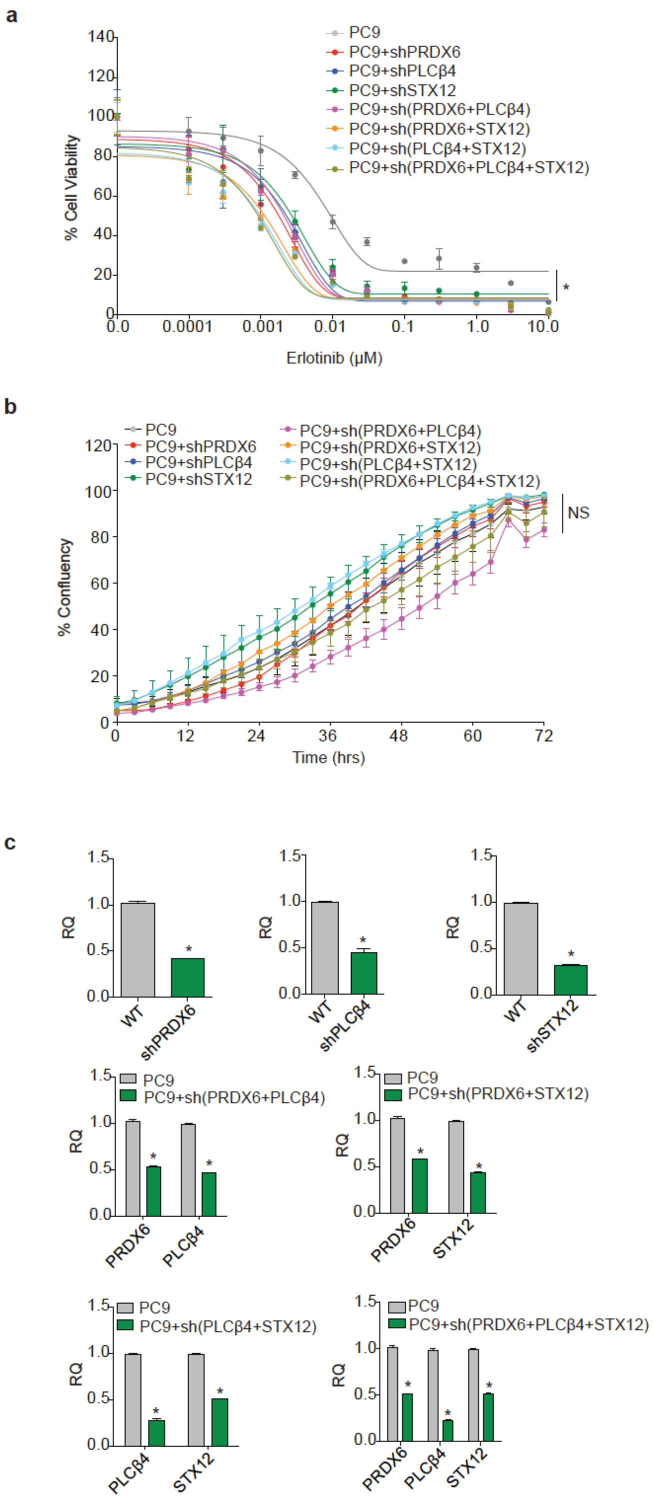

a
% Cell Viability
Erlotinib (μM)
PC9
PC9+shPRDX6
PC9+shPLCβ4
PC9+shSTX12
PC9+sh(PRDX6+PLCβ4)
PC9+sh(PRDX6+STX12)
PC9+sh(PLCβ4+STX12)
PC9+sh(PRDX6+PLCβ4+STX12)
*
b
% Confluency
Time (hrs)
NS
c
RQ
WT
shPRDX6
shPLCβ4
shSTX12
PRDX6
PLCβ4
STX12

Supplementary Figure 4. Effect of acute drug treatment on PC9 cells with stable shRNA knockdown of *PRDX6*, *PLCβ4* or *STX12* **a,** Effect on acute response to erlotinib in PC9 cells upon single, double or triple shRNA knockdown of *PRDX6*, *PLCβ4* or *STX12* was analyzed by Cell titer-Glo after 72hrs of treatment. **b,** Effect of proliferation rate of PC9 cells upon single, double or triple shRNA knockdown of *PRDX6*, *PLCβ4* or *STX12* was analyzed by Incucyte. **c,** Efficiency of shRNA knockdown of *PRDX6*, *PLCβ4* or *STX12* was analyzed by quantitative PCR of mRNA of stable PC9 cell lines. All experiments were performed in triplicate and data are representative of at least two independent experiments. Data are represented as mean ± S.E.M. * ($P< 0.05$, Student's t-test) indicates significant differences from WT controls. NS, not significant.

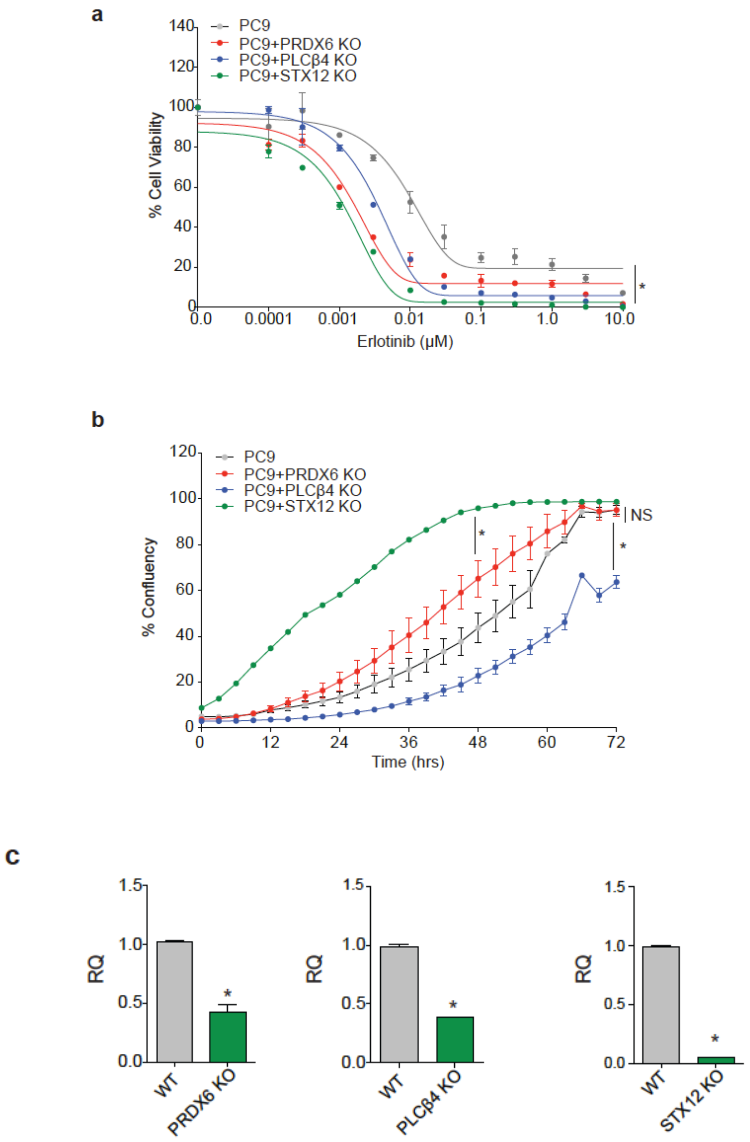

Supplementary Figure 5. Effect of acute drug treatment on PC9 cells with stable CRISPR/Cas9 knockouts of *PRDX6*, *PLCβ4* or *STX12* a, Effect on acute response to erlotinib in PC9 cells upon CRISPR/Cas9-mediated knockout of *PRDX6*, *PLCβ4* or *STX12* was analyzed by Cell titer-Glo after 72hrs of treatment. **b,** Effect of proliferation rate of PC9 cells upon CRISPR/Cas9-mediated knockout of *PRDX6*, *PLCβ4* or *STX12* was analyzed by

Incucyte. **c,** Knockout efficiency of CRISPR/Cas9-mediated knockout of *PRDX6*, *PLCβ4* or *STX12* was analyzed by qPCR of mRNA from stable PC9 cell lines. All experiments were performed in triplicate and data are representative of at least two independent experiments. Data are represented as mean ± S.E.M. * ($P < 0.05$, Student's t-test) indicates significant differences from WT controls. NS, not significant.

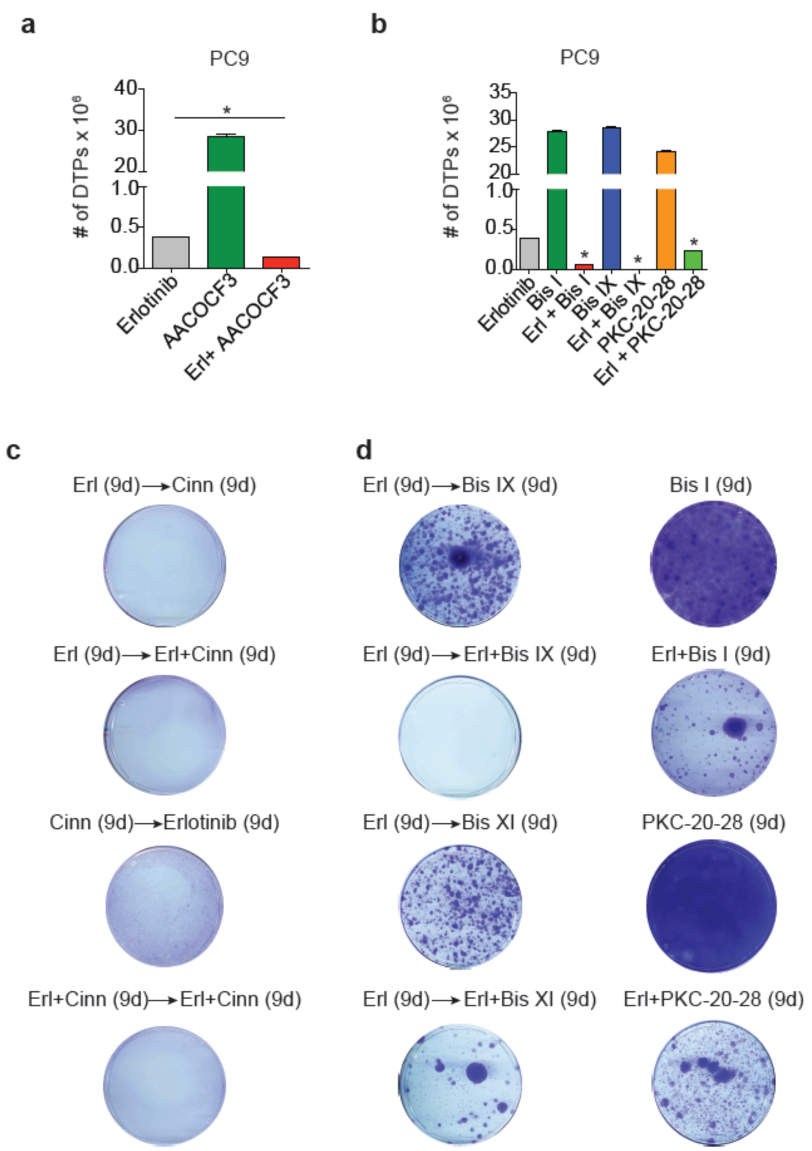

Supplementary Figure 6. Suppression of PLA2/ PKCα activity prevents formation of DTP and DTEP populations **a,** Effect of the PLA2 inhibitor AACOCF3 and **b,** PKCα inhibitors on DTP formation of PC9 cells upon co-treatment with erlotinib for 9 days (n=3). **c,** Effect of sequential treatment with erlotinib and cinnamycin for 30 days, or **d,** co-treatment or sequential treatment of erlotinib with other PKCα inhibitors for 30 days on DTEP formation of PC9 cells. All data are representative of at least two independent

experiments. Data are represented as mean ± S.E.M. * ($P < 0.05$, Student's t-test) indicates significant differences from erlotinib-treated cells.

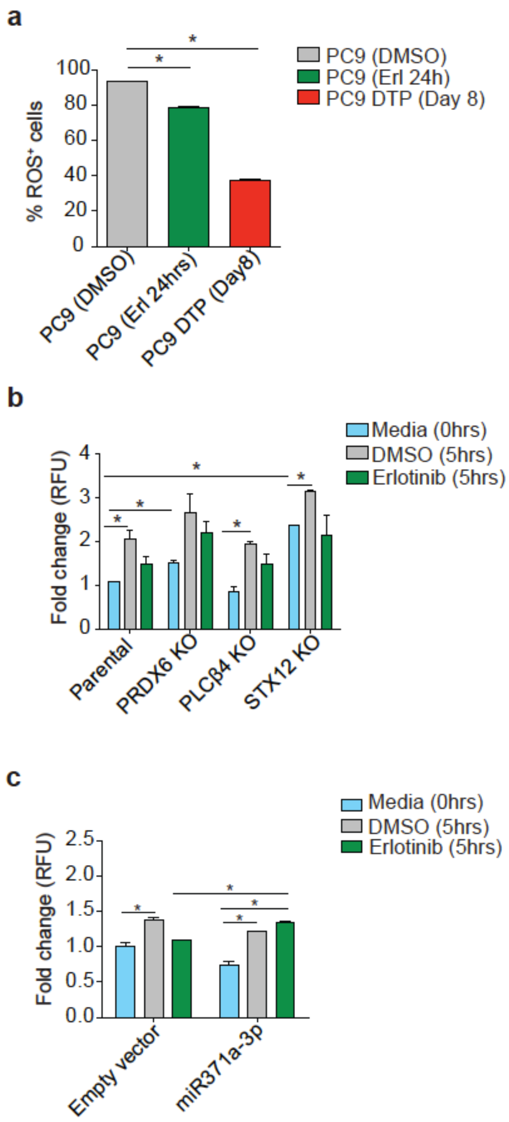

Supplementary Figure 7. Effect of miR-371-3p targets on ROS levels a, ROS levels were measured in PC9 parental and DTP cells following erlotinib treatment of parental cells for 24hrs and DTP cells for 8 days. Effect of 5hr erlotinib treatment on ROS levels in **b,** PC9 parental cells and stable *PRDX6*, *PLCβ4* or *STX12* knockout cell lines and **c,** PC9 stable cell lines expressing empty vector or pre-miR-371-3p was measured using fluorometric detection. All experiments were performed in triplicate and data are representative of at least

two independent experiments. Data are represented as mean ± S.E.M. *$P < 0.05$, Student's t-test.

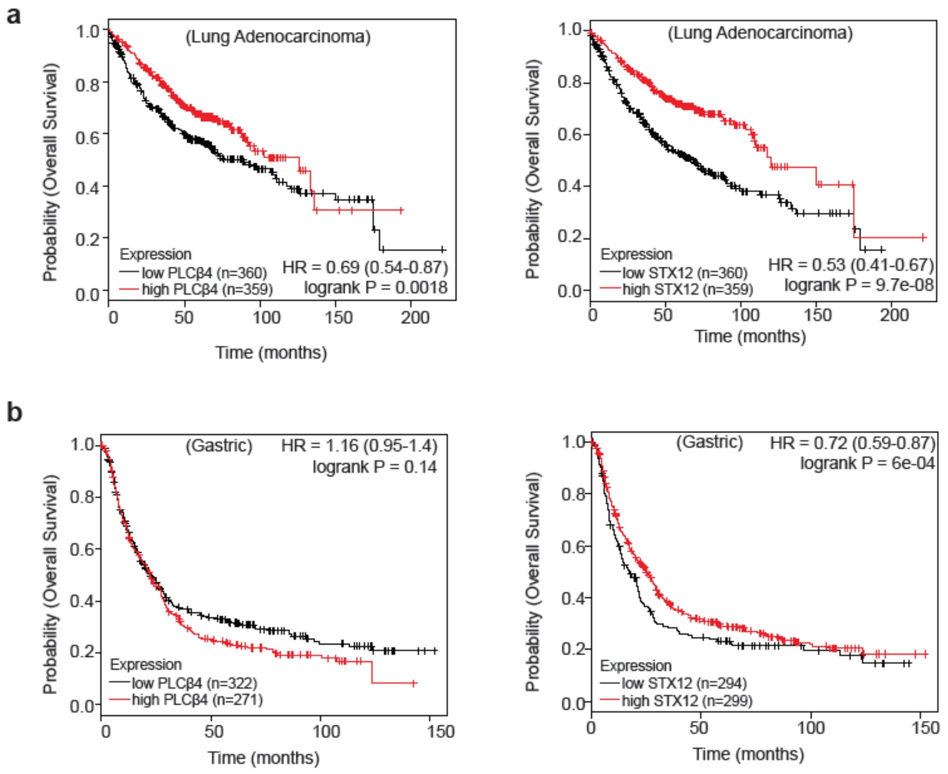

Supplementary Figure 8. Association of *PLCβ4* and *STX12* expression with clinical outcome in lung and gastric cancer subtypes **a,** Association of *PLCβ4* expression and *STX12* expression with overall survival (OS) of lung adenocarcinoma patients. The datasets used in the OS analysis were obtained from kmplot.com **b,** Association of *PLCβ4* and *STX12* expression with overall survival (OS) of gastric cancer patients. The datasets used in the OS analyses were obtained from kmplot.com.

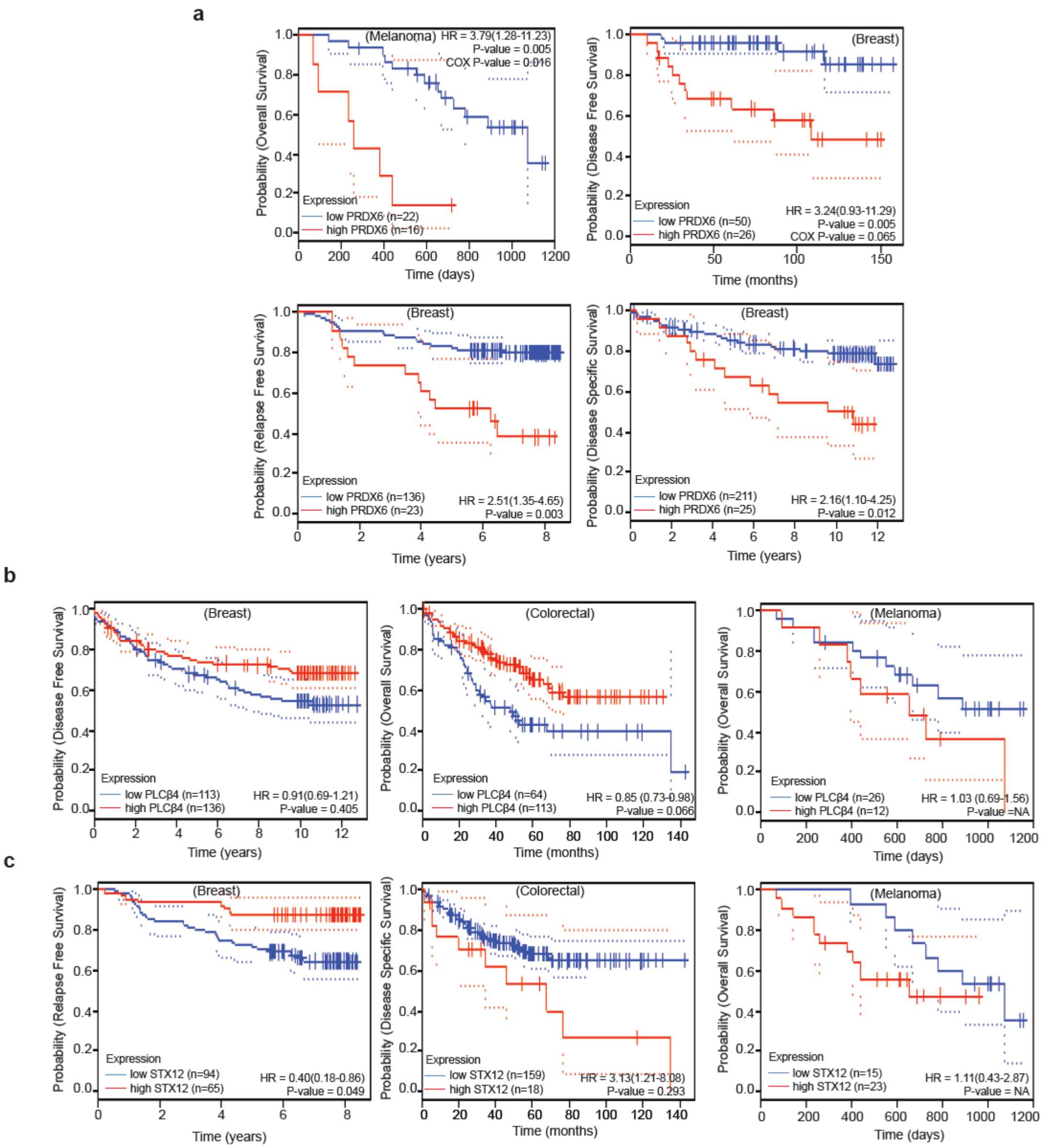

Supplementary Figure 9. Association of *PRDX6*, *PLCβ4* and *STX12* expression with clinical outcome in different cancer subtypes **a,** Kaplan-Meier plots of *PRDX6* expression and overall survival of melanoma patients (GSE19234), disease-free survival (DFS) (GSE7849), relapse-free survival (RFS) (GSE1456-GPL96) and disease-specific survival (DSS) (GSE3494-GPL96) of breast cancer patients. **b,** Association of *PLCβ4* and **c,**

STX12 expression with overall survival of breast, colorectal and melanoma patients. The datasets used for the analyses were GSE4922-GPL96 and GSE1456-GPL96 for breast cancer, GSE17536 for colorectal cancer and GSE19234 for melanoma patients.

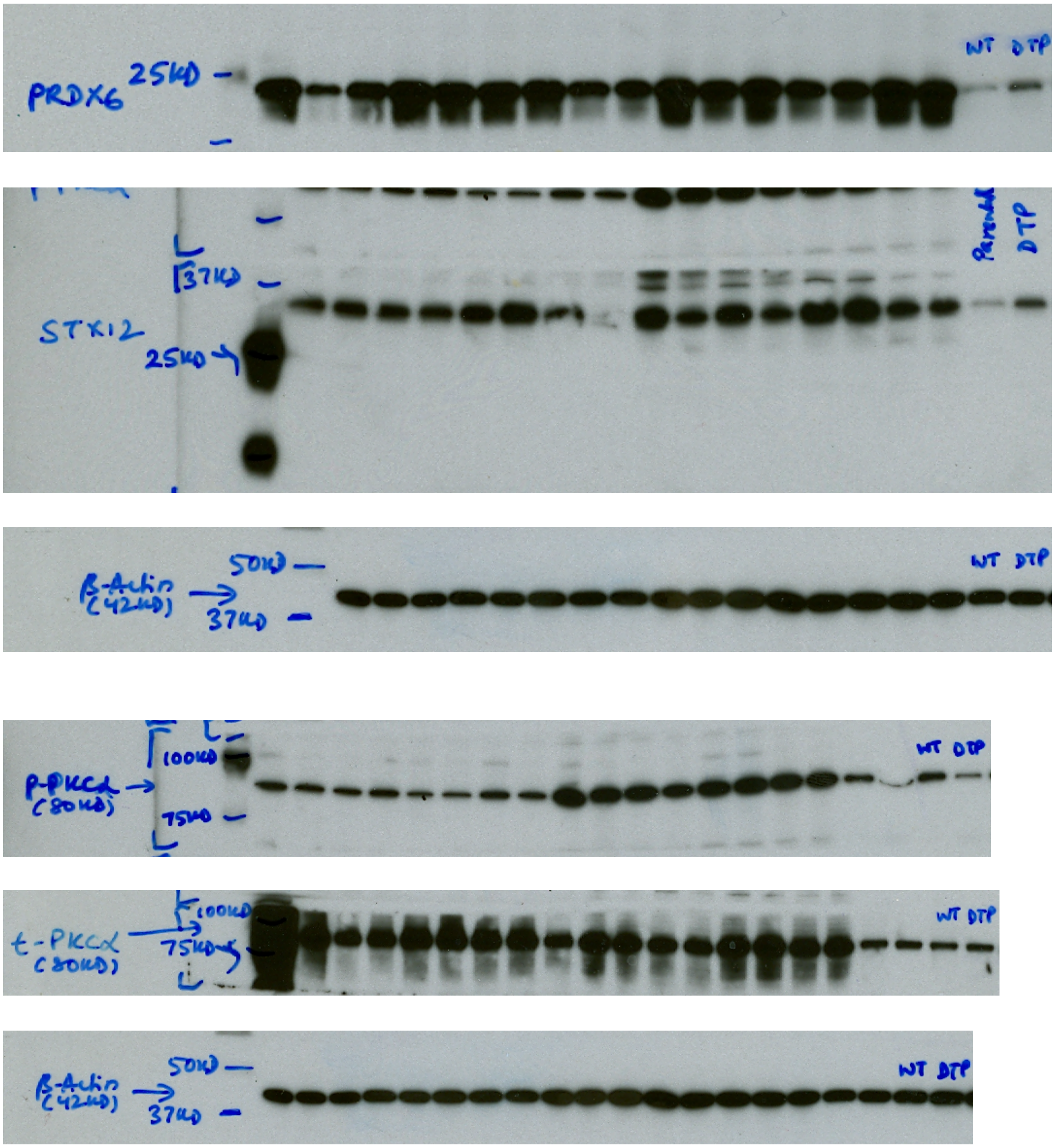
PRDX6
25KD
WT
DTP
STX12
37KD
25KD
Parental
DTP
β-Actin (42KD)
50KD
37KD
WT
DTP
p-PKCα (80KD)
100KD
75KD
WT
DTP
t-PKCα (80KD)
100KD
75KD
WT
DTP
β-Actin (42KD)
50KD
37KD
WT
DTP

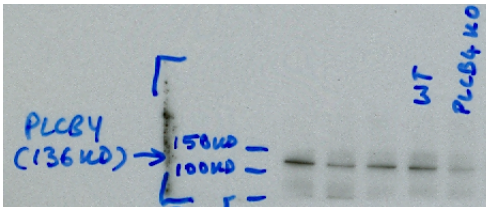
WT
PLCB4 KO
PLCB4
(136 KD)
150KD
100KD

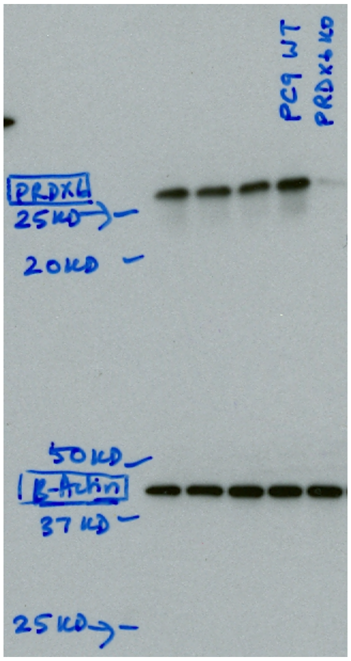
PC9 WT
PRDX6 KO
PRDX6
25KD
20KD
50KD
β-Actin
37 KD
25KD

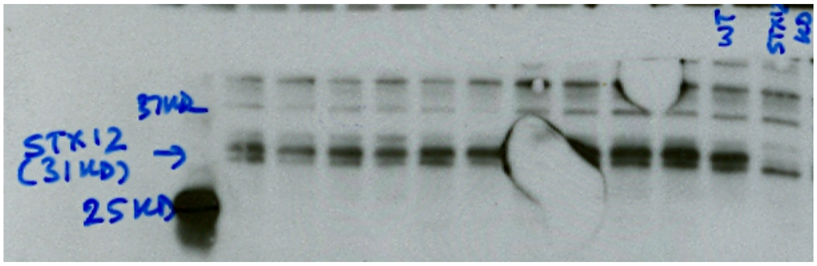
WT
STX12 KO
37KD
STX12
(31KD)
25KD

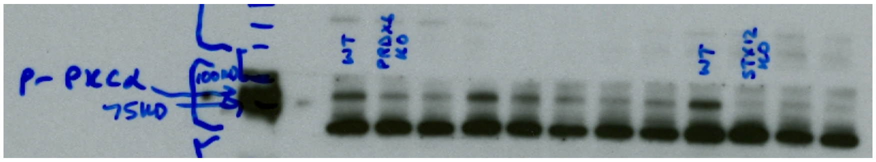
WT
PRDX6 KO
WT
STX12 KO
P-PKCα
100KD
75KD

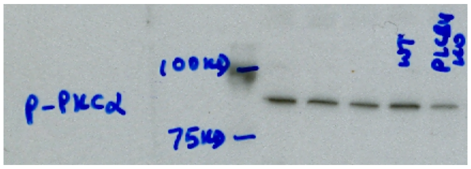
WT
PLCB4 KO
100KD
P-PKCα
75KD

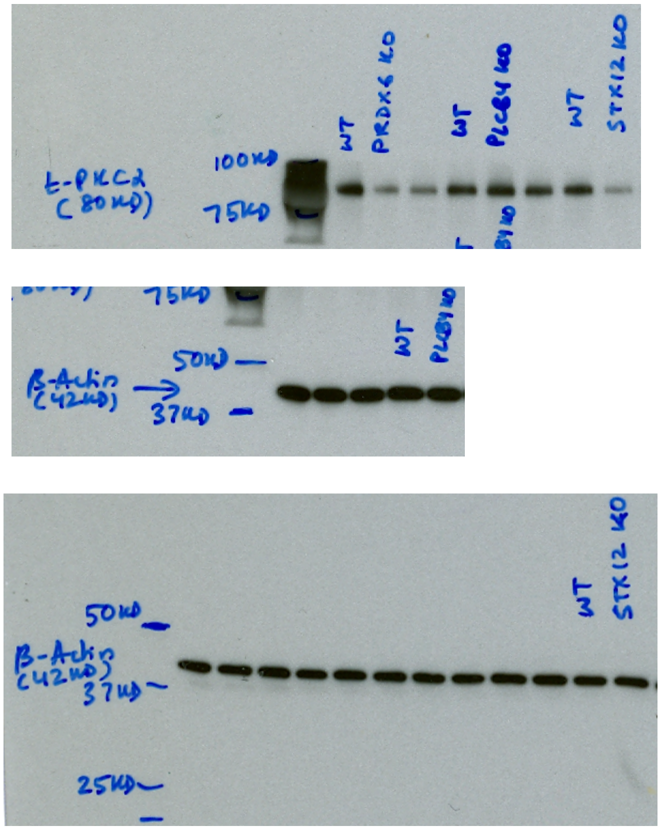

Supplementary Figure 10. Uncropped data for Figure 2h and 3f.
